# Validation of the German Benefit Finding Scale for Youth with chronic conditions

**DOI:** 10.1186/s13034-021-00438-7

**Published:** 2022-01-11

**Authors:** Roman E. von Rezori, Friederike Buchallik, Petra Warschburger

**Affiliations:** Department of Psychology, Counseling Psychology, University of Potsdam, Karl-Liebknecht- Str. 24-25, 14476 Potsdam, Germany

**Keywords:** Measure validation, Chronic conditions, Resilience, Coping skills and adjustment, Youth

## Abstract

**Background:**

Benefit finding, defined as perceiving positive life changes resulting from adversity and negative life stressors, gains growing attention in the context of chronic illness. The study aimed at examining the psychometric properties of the Benefit Finding Scale for Children (BFSC) in a sample of German youth facing chronic conditions.

**Methods:**

A sample of adolescents with various chronic conditions (*N* = 304; 12 – 21years) completed the 10-item BFSC along with measures of intra- and interpersonal resources, coping strategies, and health-related quality of life (hrQoL). The total sample was randomly divided into two subsamples for conducting exploratory and confirmatory factor analyses (EFA/CFA).

**Results:**

EFA revealed that the BFSC scores had a one-dimensional factor structure. CFA verified the one-dimensional factor structure with an acceptable fit. The BFSC exhibited acceptable internal consistency (α = 0.87 – 0.88) and construct validity. In line with our hypotheses, benefit finding was positively correlated with optimism, self-esteem, self-efficacy, sense of coherence, and support seeking. There were no correlations with avoidance, wishful thinking, emotional reaction, and hrQoL. Sex differences in benefit finding were not consistent across subsamples. Benefit finding was also positively associated with age, disease severity, and social status.

**Conclusions:**

The BFSC is a psychometrically sound instrument to assess benefit finding in adolescents with chronic illness and may facilitate further research on positive adaptation processes in adolescents, irrespective of their specific diagnosis.

## Introduction

Stress and coping research is shifting from focusing exclusively on the negative effects of chronic conditions (CCs) to an emphasis on ways in which these conditions promote positive life changes [1]. Benefit finding (BF), defined as individual differences in perceiving positive life changes resulting from adversity and negative life stressors [1, 2], herein emerged as a key construct and gained increasing attention in the context of CCs [3]. Positive life changes may manifest themselves in domains including intrapersonal benefits (e.g., feeling stronger and wiser), interpersonal benefits (e.g., feeling closer with friends and family), and changes in priorities and goals (e.g., reordering goals and emphasis of enjoyment in life) [4]. There is first meta-analytic evidence that BF in response to several health stressors is associated with lower levels of depression and global distress as well as more positive well-being [2]. While BF was studied among adults with various CCs [1, 2], studies among youth are lacking [5].

CCs are highly prevalent in youth [6] and constitute an additional challenge in their life. Transdiagnostic characteristics of CCs, namely chronicity, functional impairments, physical disability, or pain, and the need for extensive (permanent) health care, can interfere with the mastery of common developmental tasks (e.g., forming friendships, establishing first romantic relationships, school transitions or striving for autonomy and emancipation from parents) [7, 8]. So far, studies on BF in youth are limited to populations with cancer [9–11] and type 1 diabetes [12–14]. However, only one measure for BF was psychometrically evaluated for children and adolescents with cancer [11]. The Benefit Finding Scale for Children (BFSC) was adapted by pediatric clinicians from scales developed for adult patients with cancer [11]. Conducting a principal component analysis (PCA), the authors identified a single component, which accounted for 41% of the variance, and showed that the BFSC had an adequate internal consistency. Further studies on children and adolescents with cancer supported the reliability and construct validity of the BFSC (e.g., [10]).

However, it is crucial to ensure the measure provides appropriate psychometric properties when introduced to new populations [15], namely youth facing various CCs. To the best of our knowledge, this is the first study validating a BF measure for a sample of youth facing different CCs, simultaneously providing the first age-appropriate, German version. The study aimed at examining the factor structure of the BFSC, using both exploratory factor analysis (EFA) and confirmatory factor analysis (CFA). Moreover, we examined the scale’s construct validity by focusing on associations with positive intra- and interpersonal resources and coping strategies. Convergent constructs were selected based on previous reported correlates of BF, such as optimism, self-esteem, self-efficacy, empathy, acceptance, social support, and support seeking [2, 11, 16]. Discriminant constructs were chosen based on theoretical considerations. We hypothesized BF to be unrelated to measures of negative emotional reactions and passive coping strategies [17] such as cognitive avoidance, wishful thinking, and distancing oneself from the CC. Finally, we tested the BFSC against a measure of health-related quality of life (hrQoL) as an independent criterion (concurrent validity).

## Methods

### Translation process

The translation process rigorously followed the WHO guidelines [18] in order to ensure the German version of the survey was culturally valid and appropriate. With the authorization of the authors of the BFSC [11], two psychologists independently translated the BFSC into German. In an expert panel, discrepancies between both versions were discussed and a pre-final version was provided. This version was then back-translated by a bilingual person. Finally, a pilot group of youth (*N* = 5) confirmed the items were understandable.

### Procedure

Data were collected between June 2018 and August 2019 through an online questionnaire. The sample was recruited via social networks (e.g., Facebook), various self-help forums, rehabilitation facilities, and outpatient clinics in Germany. Inclusion criteria were as follows: participants’ age between 12 and 21 years, informed consent, the presence of CCs confirmed by the Children with Special Health Care Needs Screener [19], and the completion of the entire questionnaire. Participants received gift coupons (10 Euros) as incentives.

### Participants

The final sample for data analyses consisted of *N* = 304 participants aged 12 to 21 years (*M* = 17.15, *SD* = 2.80; 62.2% female). According to the MacArthur Scale [20], participants had a mean subjective social status of 6.36 (*SD* = 1.54; range = 1 – 10). Most participants, 69.4% (*n* = 211), reported to have one CC, and 30.6% (*n* = 93) reported multiple CCs. The most prominent diagnoses were endocrine, nutritional, and metabolic diseases (34.9%); diseases of the digestive system (25.3%); and diseases of the nervous system (8.9%). The mean of years since diagnosis was 6.70 years (*SD* = 5.74).

### Measures

#### Benefit finding

BF was assessed with the German translation of the BFSC [11]. Responses were recorded on a 5-point Likert scale ranging from “not at all true for me” to “very true for me”.

#### Intrapersonal resources

Psychological resources were assessed with the following six-item subscales from the “Fragebogen zur Erfassung von Ressourcen im Kindes- und Jugendalter” (FRKJ 8-16; [21]): optimism, self-esteem, self-efficacy, sense of coherence, and empathy. Respondents rated their answers on a 4-point Likert scale ranging from “never true” to “always true”. The internal consistencies in the present study (original study) were as follows: optimism: α = 0.89 (α = 0.74), self-esteem: α = 0.88 (α = 0.85), self-efficacy: α = 0.87 (α = 0.83), empathy: α = 0.83 (α = 0.83), sense of coherency: α = 0.83 (α = 0.70).

#### Coping with a disease

The Coping with a Disease Inventory (CODI; [22]) was developed to assess coping strategies in children and adolescents with CCs. The CODI consists of 28 items representing six subscales: acceptance, avoidance, cognitive-palliative coping, distance, emotional reaction, and wishful thinking. Responses were given on a 5-point Likert scale ranging from “never” to “always “. The internal consistencies in the present study (original study) were as follows: acceptance: α = 89 (α = 0.83), avoidance: α = 0.80 (α = 0.72), cognitive-palliative coping: α = 0.54 (α = 0.69), distance: α = 0.81 (α = 0.70), emotional reaction: α = 0.88 (α = 0.82), wishful thinking: α = 0.79 (α = 0.81). As the internal consistency of the subscale cognitive-palliative coping was poor, this subscale was discarded from further analyses.

#### Interpersonal resources: social support

The Berlin Social Support Scales (BSSS; [23]) were used to assess perceived social support and support seeking on a 4-point Likert scale ranging from “strongly agree” to “strongly disagree”. The internal consistencies in the present (original) study were α = 0.93 (α = 0.85) for perceived support and α = 0.87 (α = 0.81) for support seeking.

#### Health-related quality of life

The 12-item short form for the DISABKIDS chronic generic module (DCGM-12) was applied to assess general subjective hrQoL in children and adolescents with CCs [24]. The items cover mental, social, and physical hrQoL. Responses were recorded on a 5-point Likert scale ranging from “never” to “always”. As two items are referring to pharmacological treatment, and as some participants (18.1%) in our sample had no prescribed medication, we calculated the total scores for a 10-item version, too. Cronbach’s alpha of the present (original) study reached α = 0.90 (DCGM-12; α = 0.84) and α = 0.91 (DCGM-10).

#### Disease history

In addition, subjective disease severity and the age at diagnosis were assessed with single items (“I perceive my illness as severe” - 5-point Likert scale ranging from “not at all true for me” to “very true for me”; “How old were you when your illness was diagnosed by a doctor?”).

### Data analysis

The main analyses were conducted using R [25]. A two-step analytic procedure, consisting of an EFA followed by a CFA, was performed to test the factor structure [26]. First, the total dataset was split into subsamples for EFA (*n* = 100) and CFA (*n* = 204) via random sampling in IBM SPSS version 28.0 [27]. The respective sample sizes fulfilled the subject to item ratio of 10:1 and were therefore considered sufficient, given the level of the reported factor loadings < 0.50 [28] and recommendations from simulations studies (e.g., [29]). The factor structure of the BFSC was assessed in the first subsample (*n* = 100) using Ordinary Least Squared extraction (OLS). OLS is known to provide results similar to Maximum Likelihood (ML) and is considered more robust to non-normal distributed data [30]. A quartimax rotation was used, as we expected a single, orthogonal factor [30]. Factor loadings were interpreted as follows [28]: 0.71 and above excellent, 0.63 – 0.70 very good, 0.55 – 0.62 good, 0.33 – 0.45 fair, and 0.32 or lower poor.

Data from the second subsample (*n* = 204) were subjected to CFA using *lavaan* [31]. Hypothesized modeling was based on the results of the EFA in the first subsample as well as the expected one-dimensional factor structure. The CFA was performed with ML estimation with robust (Huber-White) standard errors and a scaled test statistic that is (asymptotically) equal to the Yuan-Bentler test statistic [32]. Because the χ^2^ test is sensitive to sample sizes, three indices were used to assess the model fit. An acceptable model fit was indicated by using the cut-off values of these indices: comparative fit index (CFI) of ≥ 0.90, root mean square error of approximation (RMSEA), and standardized root mean square residual (SRMR) of ≤ 0.08 were considered as acceptable [33].

As a measure of internal consistency, Cronbach’s α was calculated. In both subsamples, we examined sex differences in BF scores and correlations between BF and age, social status, disease severity, and time since diagnosis (age minus age at diagnosis). Effect sizes were calculated and interpreted by applying Cohen’s guidelines [34]: *d* = 0.20 – 0.50 representing small, *d* = 0.50 – 0.80 medium, and *d* ≥ 0.80 representing large effect sizes. Convergent as well as discriminant validity was examined via Pearson correlations with respective variables (*r* > 0.10 as small, *r* > 0.30 as medium, *r* > 0.50 as large effect size; [35]). As the level of missing data in the EFA subsample was very low (<1%), missing data were imputed using multiple imputations via fully conditional specification implemented by the MICE algorithm [36]. Multiple imputations are a robust missing data handling procedure that requires the least stringent assumptions about missing data mechanisms compared to other traditional data handling methods [37].

## Results

### Acceptance of BFSC

Nearly all participants (98.7%) missed no items on the BFSC. In total, the BFSC showed 0.2% missing data points, indicating a very low level of missing data. Little’s Test was not significant, χ2(33) = 13.72, *p* = .99, suggesting that the missing data pattern was missing completely at random.

### EFA

Means and standard deviations for all BFSC items are presented in Table 1. The data were suitable for EFA based on item distribution, average correlation to other items, and item-total correlation [38]. Bartlett’s test of sphericity indicated correlation adequacy, χ^2^(45) = 391.85, *p* < .001, and the Kaiser-Meyer-Olkin (KMO) measure indicated sampling adequacy, MSA = 0.87. The parallel analysis [39] as well as the scree plot examination [40] recommended the extraction of one factor. The results of the OLS EFA indicated that only a single factor should be extracted (λ = 4.29), explaining 42.9% of the total variance. As can be seen in Table 1, most items had good-to-excellent factor loadings except for item 4.

**Table 1:** Items of the Benefit Finding Scale, descriptive statistics, and item-factor loadings in the first sample (*n* = 100)

	Items		*M*		(*SD)*	Loading
	*Having had my illness…*					
1.	*…has helped me become a stronger person*		3.46		1.17	0.64
2.	*…has helped me learn who my real friends are*		3.19		1.50	0.63
3.	*…has helped me know how much I am loved*		3.10		1.34	0.65
4.	*…has helped me make some new best friends*		2.34		1.43	0.38
5.	*…has helped me learn to deal better with my problems*		3.02		1.24	0.62
6.	*…has helped me be more patient*		2.84		1.32	0.67
7.	*…has taught me to be more loving of others*		2.90		1.26	0.77
8.	*…has brought my family closer together*		2.89		1.39	0.52
9.	*…has taught me what is really important in life*		3.43		1.35	0.80
10.	*…has taught me to be happy and enjoy good things when they happen*		3.55		1.30	0.72

#### Further analyses

Cronbach’s alpha reached α = 0.87 (95% CI 0.83 – 0.91). There were no significant sex differences between females (*M* = 3.10; *SD* = 0.97) and males (*M* = 3.03; *SD* = 0.81), *t*(98) = − 0.39, *p* = .695, *d* = − 0.81 (95% CI − 0.49 to 0.32). BF was positively correlated with self-esteem, self-efficacy, sense of coherence, empathy, and support seeking. No significant correlations were found with hrQoL, optimism, perceived support, acceptance, avoidance, wishful thinking, distance, and emotional reaction (see Table 2). Furthermore, BF was positively associated with age and social status, but not with disease severity and time since diagnosis.

**Table 2:** Means, standard deviations and correlations between benefit finding and all measured variables in the EFA sample (*n* = 100) and CFA sample (*n* = 204)

	Correlations		*M* (*SD*)	*M* (*SD*)	Range
	(EFA)	(CFA)	(EFA)	(CFA)	
Age	0.24*	0.16*	17.02 (2.87)	17.22 (2.76)	12–21
Subjective social status	0.10	0.29***	6.49 (1.44)	6.29 (1.59)	1–10
Disease severity	0.12	0.17*	3.26 (1.03)	3.43 (1.11)	1–5
Time since diagnosis	− 0.01	0.03	7.29 (5.56)	6.41 (5.81)	0–21
Benefit Finding	–	–	3.07 (0.91)	3.16 (0.94)	1–5
Optimism	0.18	0.33**	2.74 (0.68)	2.64 (0.72)	1–4
Self-esteem	0.29**	0.27**	2.53 (0.67)	2.56 (0.67)	1–4
Self-efficacy	0.27**	0.29**	2.55 (0.59)	2.64 (0.61)	1–4
Sense of coherence	0.22*	0.27*	2.94 (0.57)	2.92 (0.62)	1–4
Empathy	0.33**	0.27**	2.98 (0.59)	2.95 (0.58)	1–4
Acceptance	0.13	0.14*	3.66 (0.90)	3.69 (0.95)	1–5
Avoidance	0.13	− 0.04	3.19 (0.98)	3.10 (1.04)	1–5
Distance	0.09	− 0.22**	2.55 (1.01)	2.40 (0.98)	1–5
Emotional reaction	− 0.05	0.14	2.60 (1.04)	2.68 (1.02)	1–5
Wishful thinking	0.04	− 0.01	3.80 (1.12)	3.80 (1.07)	1–5
Social support	0.11	0.24**	3.47 (0.67)	3.43 (0.69)	1–4
Support seeking	0.26**	0.37**	2.64 (0.76)	2.70 (0.82)	1–4
HrQoL-12^a^	− 0.04	− 0.11	3.32 (0.80)	3.22 (0.80)	1–5
HrQoL-10^b^	− 0.09	0.04	3.32 (0.85)	3.21 (0.83)	1–5

^a^ Health-related quality of life for the 12-item version. ^b^ Health-related quality of life for the 10-item version. * *p* < .05. ** *p* < .01. ***p < .001.

### CFA

**Fig. 1:**
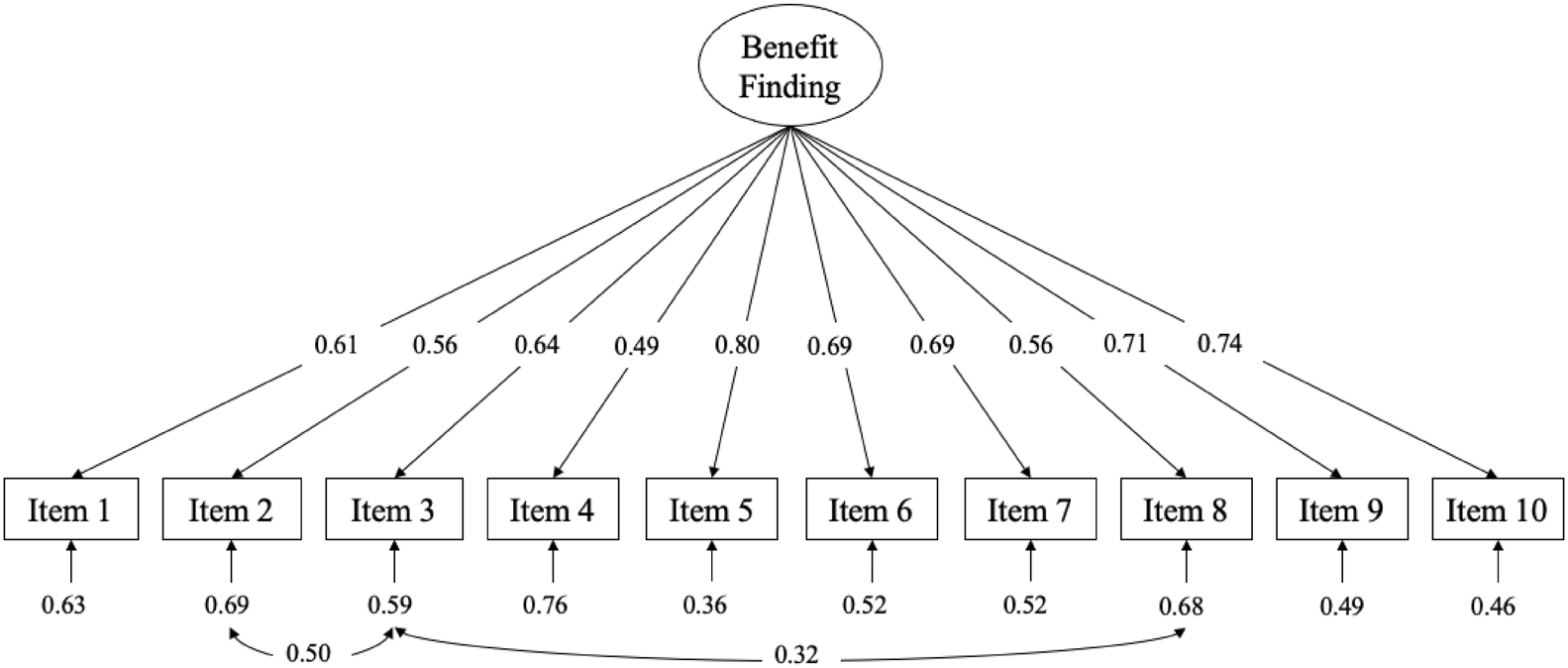
Path diagram and estimates for the one-dimensional model of the Benefit Finding Scale for Children. The large oval is the latent construct, with the rectangles representing measured variables, and the small arrow with numbers representing the residual variables (variances). The path factor loadings are standardized with significance levels were determined by critical ratios (all *p* < .001)

Based on EFA results, we examined the fit of the hypothesized one-factor solution using CFA in the second subsample. The standardized estimates of factor loadings for the best-fitting model were predominantly good-to-excellent (see Fig. 1). Item 4 showed a fair factor loading. Fit indices were as follows: RMSEA = 0.13 with 90% *CI* 0.11 – 0.16 [41], CFI = 0.86, SRMR = 0.07 and χ^2^(35) = 126.40, *p* < .001. Since the values of fit indices were not within the acceptable range, we iteratively analyzed modification indices to improve the model [42]. Two modifications were statistically meaningful (changes in model χ^2^ > 20) and theoretically plausible as they represent domains of perceived interpersonal benefits psychometrically found in a previous study [16]. Therefore, we allowed errors to correlate between item 2 (“Having my illness has helped me learn who my real friends are”) and item 3 (“Having my illness has helped me know how much I am loved”) as well as between item 3 and item 8 (“Having my illness has brought my family closer together”). After freeing two error covariances (Items 2 and 3, Items 3 and 8), the one-dimensional model provided an acceptable fit to the data: RMSEA = 0.07 with 90% *CI* 0.04 − 0.09, CFI = 0.96, SRMR = 0.05 and χ^2^(33) = 62.42, *p* = 0.001.

#### Further analyses

The internal consistency for the BFSC total score was adequate (Cronbach’s α = 0.88; 95% CI 0.86 – 0.91). Significant sex differences were observed between females (*M* = 3.29; *SD* = 0.95) and males (*M* = 2.96; *SD* = 0.90), *t*(202) = − 2.41, *p* = .02, *d* = − 0.35 (95% CI − 0.63 to 0.06). BF was significantly and positively correlated with optimism, self-esteem, self-efficacy, sense of coherence, empathy, acceptance, perceived support, and support seeking, but not with hrQoL, avoidance, wishful thinking, and emotional reaction (see Table 2). There was a significant and negative correlation between BF and distance. Moreover, BF was positively associated with age, social status, and disease severity but not with time since diagnosis.

## Discussion

The purpose of this study was to provide a German version of the BFSC [11] and to examine its psychometric properties among youth with various CCs. Previous studies have observed a one-dimensional factor structure of the BFSC in English-speaking [11] and Dutch-speaking [10] samples of children and adolescents with cancer. Our results are consistent with this literature: Using EFA, we found that all ten items of the German BFSC loaded onto the same latent dimension. Furthermore, using CFA in a second subsample, we were able to confirm that this one-dimensional model had an adequate fit following modification. Although the overall pattern of loadings was meaningful, item 4 showed only fair factor loadings, which, however, was following previous validation studies. To ensure comparability with the original study, we did not exclude this item from further analyses.

In addition, the results of our study uphold the internal consistency and construct validity of the BFSC. The BFSC showed positive correlations with a wide range of convergent constructs, while there were no significant correlations with discriminant constructs, including avoidance, wishful thinking, distance, and emotional reaction. However, it should be acknowledged that the associations between BF and acceptance, social support, and distance were not consistent across subsamples. Replicating the findings of the original study [11], the BFSC was not significantly related to hrQoL. This result highlights the notion that positive experiences (e.g., “Having had my illness has helped me to deal better with my problems”) do not simply imply an absence of negative experiences (e.g., “Does your condition get you down”) but that both rather represent independent and co-occurring dimensions. Future studies should consider alternative criteria for validation by including measures of positive well-being and satisfaction with life.

While previous studies reported no sex differences between females and males [10, 11], we observed higher scores for females, but only in our second subsample. Indeed, there is meta-analytic evidence indicating that females engage in more positive reappraisal and more positive self-talk than males [43]. This indicates that female youth might perceive higher levels of benefit in response to their CC than male youth. Studies with adequately sized samples of females and males are warranted to clarify whether BFSC scores are invariant across participant sex. Contrary to previous studies, we found that BF was positively associated with age, but not with time since diagnosis. This finding might indicate that it depends more on the developmental level and skills and does not “naturally” increase over time when coping with the disease. However, given the fact that participants of previous studies were considerably younger with mean ages around 12 years [10, 11], conclusions about the role of age and time since diagnosis should be drawn with caution. Longitudinal studies over the course of the disease including different age groups are needed to investigate BF in youth from a developmental perspective. Moreover, in line with prior work [9], our findings of CFA sample suggest that youth with CCs who report a higher subjective disease severity might be more likely to utilize BF strategies, possibly representing relevant resources of adaptive coping [44]. However, there is evidence questioning the linearity of the relation between BF and disease severity [5]. Considering research on stress-related growth, it appears there may be an inverted “U” relation, suggesting that BF experiences may be highest at moderate levels of disease severity [5]. Finally, our findings of the CFA sample indicate that youth with CCs who report higher subjective social status might be more likely to use BF strategies compared to those with lower subjective social status. While there was no significant metanalytical association between BF and socioeconomic status [2], other studies even highlight the utility of BF as a coping strategy amongst lower social status populations [45] and youth with CCs [46]. However, while previous studies only examined the association of BF with objective social status [9, 11], our study adds first insights into the link between BF and subjective social status. Evidence from prospective data indicates that subjective social status might be a more influential predictor for health status and change in health status than objective social status [47]. Further studies including both objective and subjective indicators of social status are warranted to clarify the role of subjective measures of social status in CC.

Overall, the present study had several strengths, namely the very good data quality and the sufficient sample size. Our study covered a broad age range and a wide range of underlying chronic diseases, enhancing the generalizability of our results. It should be further stressed that a methodological sound approach with an EFA-to-CFA strategy was applied, thereby overcoming the limitations of previous studies using a PCA, which is inappropriate for the identification of latent constructs and factor structures of a set of variables [48]. By focusing on intra- and interpersonal resources and coping strategies, our study provides initial evidence for potentially relevant starting points for diagnostic comparisons and transdiagnostic programs promoting BF in youth with different CCs.

Several limitations must be acknowledged. First, the recruitment strategy may have resulted in a selection bias towards generally lower levels of distress, as youth with higher levels of distress might be less likely to participate in online surveys. Second, as part of the CFA, model modifications were conducted to improve the model fit. Although modifications were based on theoretical considerations, they should be viewed as tentative until cross-validated on an independent sample [49]. Third, the cross-sectional design of our study precluded the assessment of test-retest reliability or stability of BF over time. To further strengthen the psychometric basis for the BFSC, studies with adequately-sized samples are needed to verify whether BFSC scores are invariant across group membership (e.g., sex group and diagnostic group) and measurement occasion [50]. Finally, future studies should examine whether benefit finding predicts positive adaptive outcomes, not only directly but incrementally over and above established constructs, such as emotion regulation (e.g., positive reappraisal), to further ensure the validity of BF.

## Conclusions

To conclude, the present study demonstrated that the BFSC is an economic and psychometrically sound measure that accounts for positive life changes of youths’ responses to CCs. Despite some limitations, the available evidence confirmed the one-dimensional factor structure of the BFSC also in German. This is important as it will facilitate comparison across cultures and diagnoses in future work. The application of the BFSC in future research will help to get a more comprehensive picture of the psychosocial consequences of CCs.

## Data Availability

Fully anonymized data will be available from the corresponding author on reasonable request.

## References

[1] Park CL (2009). Overview of theoretical perspectives. Medical illness and positive life change: can crisis lead to personal transformation?.

[2] Helgeson VS, Reynolds KA, Tomich PL (2006). A meta-analytic review of benefit finding and growth. J Consult Clin Psychol.

[3] Algoe SB, Stanton AL (2009). Is benefit finding good for individuals with chronic disease?. Medical illness and positive life change: can crisis lead to personal transformation?.

[4] Tedeschi RG, Calhoun LG (2004). Posttraumatic growth: conceptual foundations and empirical evidence. Psychol Inq.

[5] Meyerson DA, Grant KE, Carter JS, Kilmer RP (2011). Posttraumatic growth among children and adolescents: a systematic review. Clin Psychol Rev.

[6] van der Lee J, Mokkink L, Grootenhuis M, Heymans H, Offringa M (2007). Definitions and measurement of a systematic review. JAMA.

[7] de Ridder D, Geenen R, Kuijer R, van Middendorp H (2008). Psychological adjustment to chronic disease. Lancet.

[8] Warschburger P, Wright JD (2015). Health psychology in childhood. International encyclopedia of the social & behavioral sciences.

[9] Barakat LP, Alderfer MA, Kazak AE (2006). Posttraumatic growth in adolescent survivors of cancer and their mothers and fathers. J Pediatr Psychol.

[10] Maurice-Stam H, Broek A, Kolk AMM, Vrijmoet-Wiersma JMJ, Meijer-van den Bergh E, van Dijk EM (2011). Measuring perceived benefit and disease-related burden in young cancer survivors: validation of the Benefit and Burden Scale for Children (BBSC) in the Netherlands. Support Care Cancer.

[11] Phipps S, Long AM, Ogden J (2007). Benefit finding scale for children: preliminary findings from a childhood cancer population. J Pediatr Psychol.

[12] Helgeson VS, Lopez L, Mennella C (2009). Benefit finding among children and adolescents with diabetes. Medical illness and positive life change: can crisis lead to personal transformation?.

[13] Rassart J, Luyckx K, Berg CA, Oris L, Wiebe DJ (2017). Longitudinal trajectories of benefit finding in adolescents with type 1 diabetes. Heal Psychol.

[14] Tran V, Wiebe DJ, Fortenberry KT, Butler JM, Berg CA (2011). Benefit finding, affective reactions to diabetes stress, and diabetes management among early adolescents. Heal Psychol.

[15] Loehlin JC (2004). Latent variable models: an introduction to factor, path, and structural equation analysis.

[16] Cassidy T, McLaughlin M, Giles M (2014). Benefit finding in response to general life stress: measurement and correlates. Heal Psychol Behav Med.

[17] Compas BE, Jaser SS, Dunn MJ, Rodriguez EM (2012). Coping with chronic illness in childhood and adolescence. Annu Rev Clin Psychol.

[18] World Health Organization. Process of translation and adaptation of instruments; 2018. https://www.who.int/substance_abuse/research_tools/translation/en/.

[19] Bethell CD, Read D, Stein REK, Blumberg SJ, Wells N, Newacheck PW (2002). Identifying children with special health care needs: development and evaluation of a short screening instrument. Ambul Pediatr.

[20] Goodman Elizabeth, Adler Nancy E., Kawachi Ichiro, Frazier A. Lindsay, Huang Bin, Colditz Graham A. (2001). Adolescents' perceptions of social status: development and evaluation of a new indicator. Pediatrics.

[21] Lohaus A, Nussbeck FW (2016). FRKJ 8–16 Fragebogen zu Ressourcen im Kindes- und Jugendalter.

[22] Petersen C, Schmidt S, Bullinger M (2004). Brief report: development and pilot testing of a coping questionnaire for children and adolescents with chronic health conditions. J Pediatr Psychol.

[23] Schulz U, Schwarzer R. Soziale unterstützung bei der krankheitsbewältigung: Die Berliner Social Support Skalen (BSSS). Diagnostica; 2003.

[24] Schmidt S, Debensason D, Mühlan H, Petersen C, Power M, Simeoni MC (2006). The DISABKIDS generic quality of life instrument showed cross-cultural validity. J Clin Epidemiol.

[25] R Core Team. R: a language and environment for statistical computing. R Foundation for Statistical Computing. Vienna, Austria; 2021. https://www.r-project.org/.

[26] Worthington RL, Whittaker TA (2006). Scale development research. Couns Psychol.

[27] IBM Corp. Released 2021. IBM SPSS Statistics for Mac OS, Version 28.0. Armonk, NY: IBM Corp.

[28] Tabachnick B, Fidell L (2013). Using multivariate statistics.

[29] Mundfrom DJ, Shaw DG, Ke TL (2005). Minimum sample size recommendations for conducting factor analyses. Int J Test.

[30] Osborne JW (2014). Best practices in exploratory factor analysis.

[31] Rosseel Y. lavaan: an R package for structural equation modeling. J Stat Softw. 2012;48. 10.18637/jss.v048.i02.

[32] Muthén LK, Muthén BO. Mplus user’s guide. Los Angeles, CA Muthén Muthén; 2017.

[33] Hu L, Bentler PM (1999). Cutoff criteria for fit indexes in covariance structure analysis: conventional criteria versus new alternatives. Struct Equ Model A Multidiscip J.

[34] Cohen J (1977). Statistical power analysis for the behavioral sciences.

[35] Cohen J (1992). A power primer. Psychol Bull.

[36] van Buuren S, Groothuis-Oudshoorn K. mice: multivariate imputation by chained equations in R. J Stat Softw. 2011;45. 10.18637/jss.v045.i03.

[37] van Buuren S. Flexible imputation of missing data. 2nd edition. Boca Raton, FL: CRC Press; 2018.

[38] Clark LA, Watson D (1995). Constructing validity: basic issues in objective scale development. Psychol Assess.

[39] Horn JL (1965). A rationale and test for the number of factors in factor analysis. Psychometrika.

[40] Cattell RB (1966). The scree test for the number of factors. Multivariate Behav Res.

[41] Kline RB. Global fit testing. In: Principles and practice of structural equation modeling. 4th edition. NY: Guilford Press; 2016. p. 262–99.

[42] Whittaker TA (2012). Using the modification index and standardized expected parameter change for model modification. J Exp Educ.

[43] Tamres LK, Janicki D, Helgeson VS (2002). Sex differences in coping behavior: a meta-analytic review and an examination of relative coping. Personal Soc Psychol Rev.

[44] Rosenberg AR, Bradford MC, Barton KS, Etsekson N, McCauley E, Curtis JR (2019). Hope and benefit finding: results from the PRISM randomized controlled trial. Pediatr Blood Cancer.

[45] Chen E, Miller GE (2012). “Shift-and-persist” strategies: why being low in socioeconomic status isn’t always bad for health. Perspect Psychol Sci.

[46] Mello D, Wiebe D, Berg C (2020). Maternal shift-and-persist coping, SES, and adolescent type 1 diabetes management. Child Heal Care.

[47] Singh-Manoux A, Marmot MG, Adler NE (2005). Does subjective social status predict health and change in health status better than objective status?. Psychosom Med.

[48] Widaman KF (1993). Common factor analysis versus principal component analysis: differential bias in representing model parameters?. Multivariate Behav Res.

[49] Jackson DL, Gillaspy JA, Purc-Stephenson R (2009). Reporting practices in confirmatory factor analysis: an overview and some recommendations. Psychol Methods.

[50] Putnick DL, Bornstein MH (2016). Measurement invariance conventions and reporting: the state of the art and future directions for psychological research. Dev Rev.

